# System-wide studies of the transcriptional programming of *chromatin* during early hematopoietic development

**DOI:** 10.1186/1756-8935-6-S1-P59

**Published:** 2013-03-18

**Authors:** Nadine Obier, Mahalingam S Vijayabaskar, Stella Pearson, Maarten Hoogenkamp, Monika Lichtinger, Pierre Cauchy, David Westhead, Valerie Kouskoff, Georges Lacaud, Bertie Gottgens, Constanze Bonifer

**Affiliations:** 1 Institute of Biomedical Research, University of Birmingham, B152TT, UK; 2 Institute of Molecular and Cellular Biology, University of Leeds, LS97TF, UK; 3 Paterson Institute for Cancer Research, University of Manchester, M20 4BX, UK; 4 Cambridge Institute for Medical Research, University of Cambridge, CB2 0XY, UK

## Background

Cellular identities in multicellular organisms are defined by their individual gene expression programs. One of the great challenges for biological research is therefore to understand the molecular dynamics, hierarchy and interdependencies of transcriptional regulation leading to cell fate decisions in the course of differentiation and development.

## Materials and methods

Here we use *in vitro* differentiation of pluripotent mouse embryonic stem (ES) cells to macrophages as a model to identify molecular mechanisms and dynamics of transcriptional programs that define the individual cellular identities. We analyze the chromatin landscape and gene expression of the following five successive cell types that are purified by FACS according to their specific surface marker profiles: mesoderm, hemangioblast, hemogenic endothelium, hematopoietic precursors and macrophages. Genome-wide transcriptional activity of the individual cell populations was assessed by RNA sequencing and the distribution of histone modifications associated with either active or repressed regulatory elements was investigated by ChIP sequencing. Moreover, general DNA accessibility, i.e. open chromatin structure, was detected by DNase 1 digestion and subsequent genome-wide sequencing.

## Results

So far we started analyzing hematopoietic precursor cells. As expected, we found a strong correlation between the levels of gene expression and histone modifications associated with active chromatin, such as H3K4me3, around transcriptional start sites (TSSs) of genes. Also, the DNase 1 accessibility at TSSs directly correlated with increasing transcriptional activity. However, we found elevated levels of the Polycomb-mediated repressive H3K27me3 mark only around TSSs of genes with low expression but not around TSSs of genes with high, medium or no expression. Further, in hematopoietic precursor cells we observed an enrichment of H3K27me3 around TSSs of such genes that 1) would be repressed in the next differentiation stage, 2) are transiently repressed but were active before and would be active in the next subsequent differentiation stage, and 3) are just being repressed from this differentiation stage onwards. In contrast, genes not changing their expression levels were not associated with H3K27me3 at all.

## Conclusion

The first glimpse we got of our data reveals interesting insights into the interplay between the repressive H3K27me3 modification at TSSs and gene repression. Our further analyses will include the integration of data of all five cell types and the investigation of not only promoters but mainly enhancers. By this we aim to address the question of how the ordered, hierarchical and dynamic interplay of transcription factors and specific chromatin states eventually lead to the stable expression of lineage specific genetic programs.

